# Extensive translation of circular RNAs driven by *N*^6^-methyladenosine

**DOI:** 10.1038/cr.2017.31

**Published:** 2017-03-10

**Authors:** Yun Yang, Xiaojuan Fan, Miaowei Mao, Xiaowei Song, Ping Wu, Yang Zhang, Yongfeng Jin, Yi Yang, Ling-Ling Chen, Yang Wang, Catherine CL Wong, Xinshu Xiao, Zefeng Wang

**Affiliations:** 1 Institute of Biochemistry, College of Life Sciences, Zhejiang University at Zijingang, Zhejiang, Hangzhou, Zhejiang 310058, China; 2 CAS Key Lab for Computational Biology, CAS Center for Excellence in Molecular Cell Science, CAS-MPG Partner Institute for Computational Biology, Shanghai Institute for Biological Sciences, Chinese Academy of Sciences, Shanghai 200031, China; 3 Department of Integrative Biology and Physiology and the Molecular Biology Institute, UCLA, Los Angeles, CA 90095, USA; 4 Department of Pharmacology, University of North Carolina at Chapel Hill, Chapel Hill, NC 27599, USA; 5 Synthetic Biology and Biotechnology Laboratory, State Key Laboratory of Bioreactor Engineering, School of Pharmacy, East China University of Science and Technology, Shanghai, China; 6 National Center for Protein Science, Institute of Biochemistry and Cell Biology, Shanghai Institutes for Biological Sciences, Chinese Academy of Sciences, Shanghai 200031, China; 7 Shanghai Science Research Center, Chinese Academy of Sciences, Shanghai 201204, China; 8 Institute of Biochemistry and Cell Biology, Shanghai Institute for Biological Sciences, Chinese Academy of Sciences, Shanghai 200031, China; 9 Institute of Cancer Stem Cell, Dalian Medical University, Dalian, Liaoning 116044, China

**Keywords:** *N*^6^-methyladenosine, circular RNA, cap-independent translation, eIF4G2

## Abstract

Extensive pre-mRNA back-splicing generates numerous circular RNAs (circRNAs) in human transcriptome. However, the biological functions of these circRNAs remain largely unclear. Here we report that *N*^6^-methyladenosine (m^6^A), the most abundant base modification of RNA, promotes efficient initiation of protein translation from circRNAs in human cells. We discover that consensus m^6^A motifs are enriched in circRNAs and a single m^6^A site is sufficient to drive translation initiation. This m^6^A-driven translation requires initiation factor eIF4G2 and m^6^A reader YTHDF3, and is enhanced by methyltransferase METTL3/14, inhibited by demethylase FTO, and upregulated upon heat shock. Further analyses through polysome profiling, computational prediction and mass spectrometry reveal that m^6^A-driven translation of circRNAs is widespread, with hundreds of endogenous circRNAs having translation potential. Our study expands the coding landscape of human transcriptome, and suggests a role of circRNA-derived proteins in cellular responses to environmental stress.

## Introduction

Although circular RNAs (circRNAs) in higher eukaryotes were first discovered more than 20 years ago^1,2^, they attracted little attention until recently when a large number of circRNAs were identified by parallel sequencing^3,4,5,6,7^. The majority of circRNAs are generated through back-splicing of internal exons, a non-canonical splicing process promoted by dsRNA structures across circularizing exons^3,8,9,10,11^. Although there are reports that some circRNAs can function as “decoys” to neutralize miRNA (i.e., as miRNA sponges)^4,7^ or to bind and sequester other RNA binding proteins^12^, the biological function of most circRNAs is still undetermined. An intriguing possibility is that circRNAs could be translated to produce proteins, because most of circRNAs originate from exons and are localized in the cytoplasm. Indeed, artificial circRNAs with an internal ribosomal entry site (IRES) can be translated *in vitro*^13^ or *in vivo*^11^. However the coding potential of circRNAs remains an open question because of early reports that most circRNAs are not associated with polysomes^3,14,15^.

*N*^6^-methyladenosine (m^6^A) is the most abundant internal modification of RNAs in eukaryotes^16,17^. The modification preferably occurs in the consensus motif “RRm^6^ACH” (R = G or A; H = A, C or U)^18,19^, and is found in over 7 000 mRNAs and 300 non-coding RNAs in human and mouse using m^6^A-specific immunoprecipitation (MeRIP-Seq)^20,21^. The methylation of adenosine is catalyzed by a methyltransferase complex consisting of methyltransferase-like 3 (METTL3), METTL14 and Wilms' tumor 1-associating protein^22,23,24,25^, and the m^6^A is demethylated by fat mass and obesity-associated protein (FTO) and alkylated DNA repair protein alkB homolog 5 (AKLBH5)^26,27^, which serve as the writers and erasers of m^6^A. Inside cells, m^6^A is specially recognized by the YTH domain family protein YTHDF1, 2 and 3 that bind m^6^A and function as m^6^A readers. The m^6^A modification can affect multiple stages of RNA metabolism, including mRNA localization, splicing, translation and degradation, which in turn regulates important biological processes such as stem cell differentiation^28,29^. In particular, m^6^A is reported to have multifaceted affects on translation: m^6^A in 3′ UTRs was found to increase translation efficiency through binding of YTHDF1^30^, whereas m^6^A in 5′ UTRs was reported to promote cap-independent translation upon heat shock stress through a YTHDF2-protection mechanism^31,32^. In addition, a recent study has reported that m^6^A reader YTHDF3 promotes protein synthesis in synergy with YTHDF1^33^, which is further supported by the finding that cytoplasmic YTHDF3 interacts with ribosomal proteins to promote mRNA translation^34^. However the general effect of m^6^A on protein translation is still an incomplete story with the detailed mechanisms being elusive.

Here we report that circRNAs in human cells can be efficiently translated using short sequences containing m^6^A site as IRESs. Consistently, the translation from circRNA is reduced by m^6^A demethylase FTO, promoted by adenosine methyltransferase METTL3/14, and requires eukaryotic translation initiation factor eIF4G2 and m^6^A reader YTHDF3. We found that a large number of circRNAs are methylated, suggesting that such translation could be common to many circRNAs. By sequencing RNase R-resistant RNAs associated with polysomes, we identified hundreds of endogenous translatable circRNAs, many of which contain m^6^A sites. Our finding suggests a general function of circRNA and has important implications in the translation landscape of human genome.

## Results

### circRNAs containing m^6^A motifs are translated inside cells

**Figure 1:**
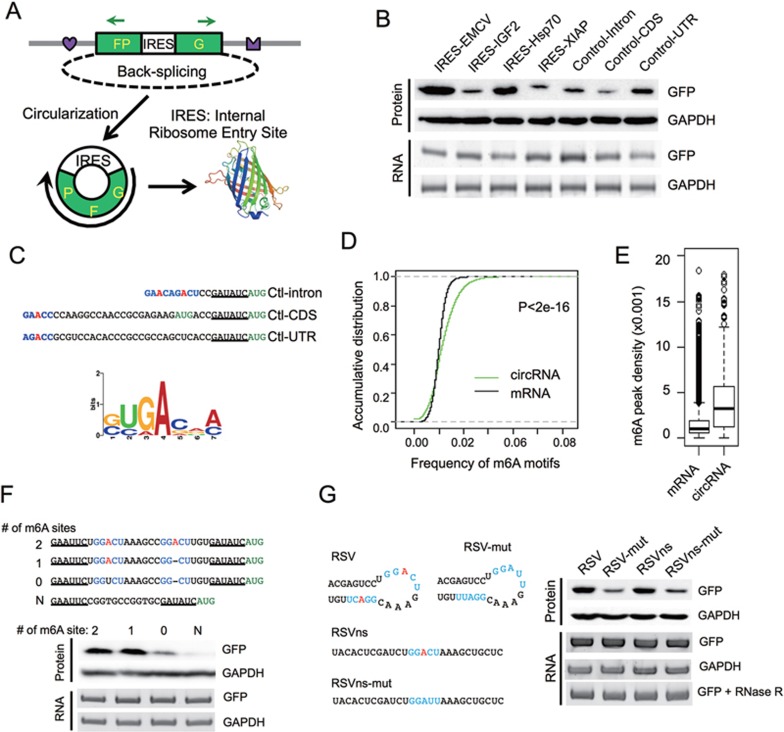
*N*^6^-methyladenosine promotes circRNA translation. **(A)** Schematic diagram of a circRNA translation reporter consisting of a single exon and two introns with complement sequences (marked by heart and crown). The exon can be back-spliced to generate circRNAs that drive GFP translation from the IRES. Green arrows indicate PCR primers used in detecting circRNA. **(B)** Translation of circRNA can be driven by different endogenous human IRESs (from IGF2, Hsp70 and XIAP) or three control sequences (short fragments of intron, coding region and 5′ UTR from beta-Actin gene, see Supplementary information, Table S1). Each reporter was transfected into 293 cells, and protein production was detected by western blot 48 h after transfection. CircRNA was detected by semi-quantitative PCR using circRNA specific primers. **(C)** Consensus m^6^A motifs are enriched in the “negative control” sequences. Top, RRACH motif near the start codon, with the putative m^6^A-modified adenosine highlighted in red and the start codon labeled in green. Bottom, enriched motifs discovered by k-mer sampling/clustering. **(D)** Accumulative distribution of m^6^A motif in circRNA and mRNA. **(E)** Density of m^6^A peaks (from MeRIP-seq) that are mapped to all mRNAs and known circRNAs regions. *P*-value = 3.2e^−7^ by student's *t*-test. **(F)** m^6^A motifs directly promote circRNA translation. 0-2 copies of m^6^A motifs (GGACU) and an adenosine-free control sequence (CGGTGCCGGTGC) were inserted into the upstream of the start codon in circRNA reporters, and circRNA and GFP translation were detected similarly as in panel **B**. **(G)** Known m^6^A sites (RSV and RSVns) and their mutations were tested for the activity of driving translation. Experimental procedures are the same as in panel **B**. In last panel, the total RNA was also treated with RNase R before RT-PCR.

Previously we developed a minigene reporter containing split GFP to demonstrate that a circRNA can be translated using a viral IRES^11^ (Figure 1A). The translation from this circRNA system was rigorously validated by multiple controls, including introducing mutations that disrupt intron pairing, treating the samples with RNase R and cleaving the expression plasmids into linear DNA with restriction enzymes to eliminate potential artifact of exon concatemers^11^. To further explore the molecular mechanisms governing this phenomenon, we analyzed whether the circRNAs containing several other endogenous IRES or control sequences in human genome can also be translated (Supplementary information, Table S1). Surprisingly, all the inserted sequences, including the three “negative controls” ranging from 38 to 253 nt, efficiently initiated GFP translation as judged by western blots (Figure 1B) and fluorescence microscopy (Supplementary information, Figure S1A). Translation from the circRNA was eliminated only when the sequence of restriction sites was left between the stop and start codons of GFP in the circRNA reporter (Supplementary information, Figure S1B). This unexpected result suggested that IRESs serving to facilitate translation initiation in circRNAs are more prevalent than previously expected.

To understand how the “negative control” sequences induce circRNA translation, we examined their sequences near the translation start site. Interestingly, all “negative control” sequences contain a RRACH fragment (R = G or A; H = A, C or U) near the start codon (Figure 1C, top), resembling the consensus motif of m^6^A modification (i.e., RRACH motif), the most abundant internal modification of RNAs^16,17^. Moreover, by clustering the randomly sampled 10-nt sequence windows in these three sequences, we also found a motif that resembles the consensus site for m^6^A modification (Figure 1C, bottom, see Materials and Methods section). Compared to all coding mRNAs, the putative m^6^A motifs are significantly enriched in known circRNAs (Figure 1D). This observed enrichment is reliable, because in a control analysis, m^6^A motifs are more enriched in snoRNAs yet depleted in snRNAs^35^, and are more enriched in exons compared to introns as expected (Supplementary information, Figure S1C). Furthermore, compared to randomly selected 1 000 sets of control hexamers, the consensus m^6^A hexamers (i.e., HRRACH) are significantly enriched in known circRNAs (Supplementary information, Figure S1D). Consistently, we found that previously identified m^6^A peaks from transcriptome-wide mapping of m^6^A sites using MeRIP-Seq^20,21^ have a higher average density in circRNA regions compared to all mRNAs regardless of the relative positions (Figure 1E).

Because m^6^A was recently found to increase mRNA translation efficiency^30,32^, our observations strongly suggested that the m^6^A containing RRACH sequences may be involved in the translation initiation of circRNAs. To test this hypothesis, we inserted a short fragment (19 nt) containing different copies of consensus m^6^A motifs before the start codon of circRNA reporter and measured GFP protein production in transfected 293 cells (Figure 1F). As expected, circRNAs containing one or two m^6^A motifs were efficiently translated into GFP protein, whereas the mutation of both motifs greatly reduced (but did not completely eliminate) the GFP level (Figure 1F). In addition, the circRNA with single m^6^A site has similar translation efficiency compared to circRNA with two m^6^A sites, indicating that a single m^6^A site is sufficient to initiate translation (i.e., excessive m^6^A modification may not increase circRNA translation efficiency). The translation of GFP was eliminated when we inserted a sequence without any adenosine residue (Figure 1F). In addition, we tested two other sequences (RSV and RSVns) that were reported to undergo m^6^A modification^23^, and found that both sequences strongly induced protein translation. Importantly, mutants that decrease m^6^A methylation in these sequences^23^ also reduced the translation efficiency (Figure 1G), further supporting the notion that m^6^A drives protein translation from circRNAs. The production of circRNAs from all reporters was further validated with northern blot using a probe that specifically recognizes full-length GFP (Supplementary information, Figure S2A).

As previously reported, the circRNA and its translation products were detected even after the linearization of reporter plasmids with *Mlu*I digestion (Supplementary information, Figure S2B), strongly supporting translation from circRNA because the pre-mRNA with multiple copies of concatenated GFP fragments cannot be produced in this scenario. The same set of sequences was also able to drive protein translation in HeLa cells (Supplementary information, Figure S3A and S3B), indicating that m^6^A-initiated translation is cell type independent. However in HeLa cells, there is still some expression of GFP following mutation of both m^6^A motifs (Supplementary information, Figure S3A). This might be because either m^6^A can occur at a non-canonical site or some sequences can initiate translation in m^6^A-independent fashion in HeLa cells.

### Modulation of m^6^A level in circRNA affects translation efficiency

**Figure 2:**
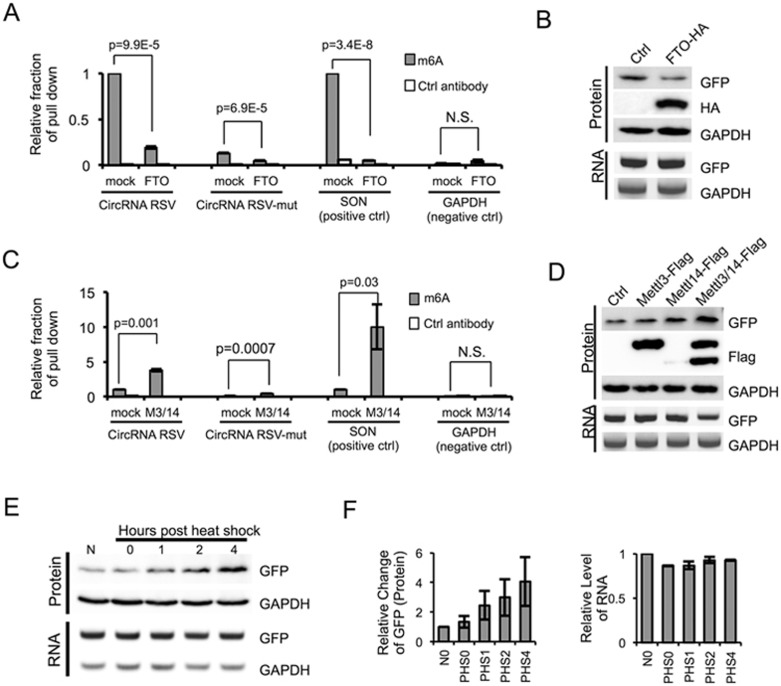
Methylation of circRNA affects translation efficiency. **(A)** m^6^A in circRNA is reduced by FTO. FTO expression vector was co-transfected with circRNA containing RSV or RSV-mut m^6^A site into 293 cells, and the RNAs from transfected cells were pulled down by m^6^A-specific antibody and analyzed by RT-qPCR. The SON mRNA known to contain multiple m^6^A sites and GAPDH mRNA containing no m^6^A modification were used as controls. Control antibody is anti-GAPDH antibody. The IP experiments were repeated three times, with mean and SD plotted. **(B)** FTO reduces circRNA translation. RNA and protein were analyzed by semi-quantitative RT-PCR and western blots using 293 cells transfected with circRNA reporter containing RSV and FTO (or mock control). **(C)** METTL3 and METTL14 can methylate circRNA. circRNA with RSV or RSV-mut, METTL3 and METTL14 overexpression plasmids were co-transfected into 293 cells as in **A** (*n* = 3; mean ± SD). **(D)** circRNA translation is increased by METTL3/14. Experimental procedures are the same as in **B**. **(E)** 293 cells transiently expressing circRNA with RSV were subjected to heat shock stress. Cells were collected at 0, 1, 2, 4 h after heat shock (1 h at 42 °C) to analyze RNA and protein expression using semi-quantitative RT-PCR and western blots. N, no heat shock. **(F)** Quantification of circRNA RNA and GFP protein levels in heat-shocked cells. GAPDH levels were used for normalization (*n* = 3, mean ± SD).

To further evaluate the importance of m^6^A in translation of circRNAs, we examined whether circRNAs with m^6^A motifs are indeed methylated using RNA-IP. We found that antibody against m^6^A specifically pulled down circRNA containing the RSV m^6^A site and a known m^6^A-containing mRNA (SON mRNA)^20^, but not a control mRNA without m^6^A (GAPDH) (Figure 2A). CircRNA containing mutated m^6^A site (RSV-mut) was also pulled down by m^6^A antibody, but with dramatically reduced efficiency (Figure 2A). This observation suggests that the mutation can reduce but not completely eliminate the m^6^A modification at RSV site, which is consistent with the small amount of GFP production in RSV-mut reporter (Figure 1G). Alternatively, there may be other minor sites for m^6^A modification in the circRNA. In addition, co-expression of m^6^A demethylase FTO^26^ significantly reduced the abundance of immunoprecipitated SON mRNA or RSV-containing circRNA (Figure 2A) and decreased the translation of GFP from the circRNA (Figure 2B), further confirming that the circRNA/mRNA with m^6^A sites are methylated and circRNA translation is indeed driven by m^6^A. In agreement with these observations, co-expression of m^6^A methyltransferase METTL3/14 significantly increased the RNA-IP signal from the circRNA or mRNA containing m^6^A but not from the control RNA (Figure 2C), and greatly increased protein translation from circRNA (Figure 2D). Interestingly, expression of METTL14 is unstable by itself but the co-expression of METTL3 greatly stabilized METLL14 and synergistically induced the translation of the GFP protein, supporting a previous report that METTL3 and METLL14 may form a stable complex^23^ (Figure 2D). We also noticed that expression of FTO or METTL3/14 did not change the level of circRNA, suggesting that m^6^A modification has little effect on the stability of circRNA. This observation differs from the report that m^6^A modification promotes degradation of linear mRNA^36^, and probably reflects a greater stability of circRNAs so that the small change of stability resulting from m^6^A modification is not as obvious, or because degradation of circRNA is regulated by a different mechanism compared to linear mRNA.

*N*^6^-methylation of adenosine has been shown to affect mRNA translation under heat shock stress^31,32^. In line with this idea, translation of GFP protein from the m^6^A-containing circRNA increased in a time-dependent manner during the 37 °C recovery phase following 1-h treatment at 42 °C (Figure 2E and 2F), while the levels of the GFP circRNA remaining unchanged (Figure 2E and 2F). This finding suggests that m^6^A-mediated protein translation, particularly from circRNAs, may be an important element in cellular stress responses. One possible mechanism by which heat shock stress could enhance circRNA translation is by translocation of YTHDF2 from cytosol into nucleus upon heat shock to block the m^6^A “eraser” FTO^31^, thus increasing the level of m^6^A modification in circRNAs. Alternatively, heat shock stress can reduce cap-dependent translation globally and cause cells to shift toward cap-independent translation through IRESs (reviewed by Spriggs *et al*.^37^); thus cap-independent translation from circRNA would be increased accordingly.

### Protein factors required for m^6^A-initiated circRNA translation

**Figure 3:**
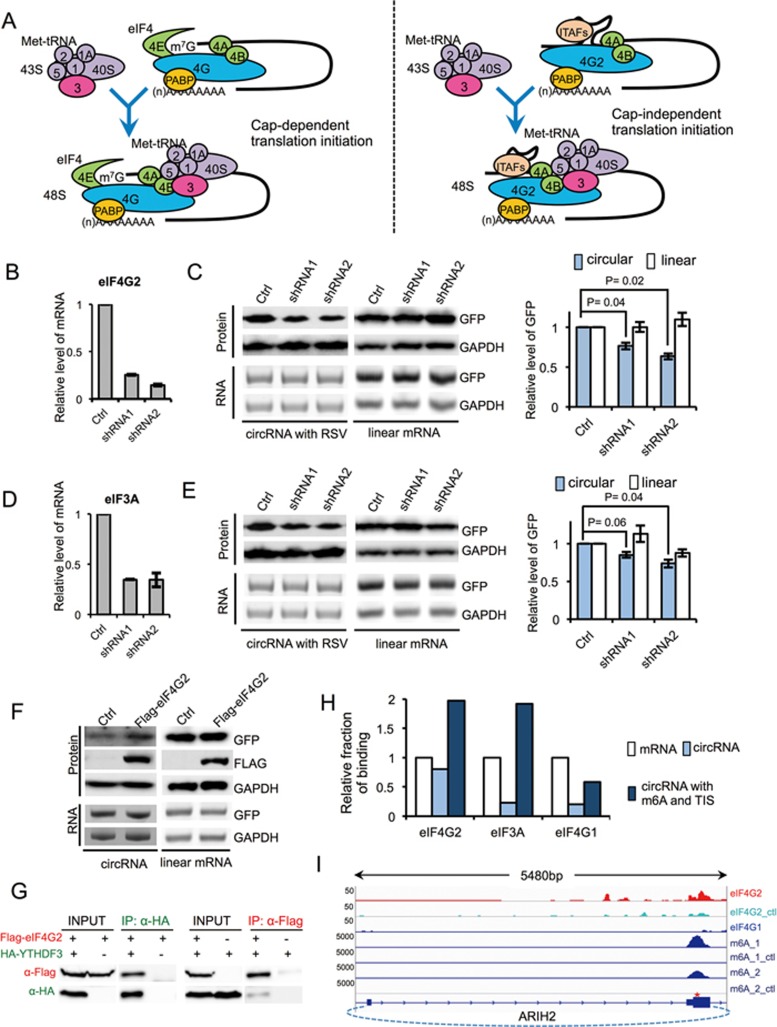
Initiation factors eIF3A and eIF4G2 affect circRNA translation. **(A)** Schematic diagram of cap-dependent and cap-independent translation initiation in eukaryotic cells. In cap-dependent translation, eIF4 complex recognizes m^7^G and recruits 43S complex to mRNA to initiate translation. In cap-independent translation, eIF4G2 directly binds to the mRNA and recruitments 43S complex to mRNA to initiate translation. **(B)** eIF4G2 knockdown by two different shRNAs stably expressed in 293 cells. **(C)** RNAi of eIF4G2 decreases circRNA translation. 293 cells stably expressing shRNAs were transfected with circRNA reporters containing RSV sequence or linear GFP reporters (pEGFP-C1). RNA and protein expression levels were analyzed by semi-quantitative RT-PCR and western blots (left). Quantification of GFP protein levels was normalized to GAPDH (right; *n* = 3, mean ± SD). **(D)** eIF3A knockdown by two different shRNAs stably expressed in 293 cells. **(E)** RNAi of eIF3A decreases circRNA translation. Experimental procedures are same as in **C** (*n* = 3, mean ± SD). **(F)** eIF4G2 overexpression increases the circRNA translation. circRNA with RSV and eIF4G2 overexpression plasmids were co-transfected into 293 cells, and the levels of proteins and circRNAs were detected with western blots and RT-PCR. **(G)** Expression vectors of eIF4G2 and YTDHF3 with different epitope tags were co-expressed in 293 cells, and the anti-Flag or anti-HA antibodies were used for precipitation. **(H)** Relative fraction of eIF4G2, eIF3A and eIF4G1-binding sites in mRNAs, circRNAs and circRNAs with m^6^A site and translation initiation site. **(I)** eIF4G2 binding site and m^6^A peak in circular ARIH2 RNA.

CircRNA translation needs to be initiated through a mechanism fundamentally different from linear mRNA that is initiated by ribosomal scanning. Eukaryotic translation is initiated by eIF4 complex^38^, of which eIF4E binds to the mRNA cap and eIF4G serves as a protein-binding scaffold to assemble the initiation complex (Figure 3A). Activated 40S ribosomal subunit is subsequently recruited to mRNA through binding of eIF3 to eIF4G^38^. In the cap-independent translation, a non-canonical eIF4G protein (eIF4G2) directly recognizes an IRES to initiate eIF4 complex assembly in the absence of eIF4E (Figure 3A), leading to translation initiation^39^. Therefore, to further understand m^6^A-driven translation of circRNAs, we investigated the possible involvement of eIF4G2 in the translation initiation of circRNAs. Using stable cell lines expressing two shRNAs against eIF4G2 (Figure 3B), we examined the expression of GFP encoded by either circRNA or linear mRNA. As expected, eIF4G2 depletion significantly reduced protein translation from circRNA but had no effect on translation from linear mRNA (Figure 3C). Similarly, depletion of eIF3A, an eIF3 subunit bound to viral IRES^40^, modestly reduced protein translation from circRNA but did not affect linear mRNA translation (Figure 3D and 3E). This result is consistent with previous finding that eIF3A is involved in the m^6^A-promoted translation^32^. As expected, depletion of eIF4G2 by RNAi had little effect on global protein translation rate, whereas eIF3A depletion significantly reduced global protein synthesis (Supplementary information, Figure S4A-S4B). This result is consistent with that eIF4G2 knockdown has more obvious effect in reducing protein translation from circRNA. We further confirmed that the overexpression of eIF4G2 indeed increased GFP translation from circRNA but not from linear mRNA by co-expressing eIF4G2 with the circRNA or linear mRNA encoding for GFP (Figure 3F). Collectively, these results suggest that the translation of circRNA may be initiated by an eIF4G2-dependent mechanism similar to other IRESs.

We next examined whether the m^6^A reader proteins are required in the translation of m^6^A-containing circRNAs. We found that depletion of YTHDF1 by RNAi did not affect translation from circRNA (Supplementary information, Figure S5A-S5B), and YTHDF2 depletion slightly inhibited GFP translation from both circRNA and linear RNA (Supplementary information, Figure S5C and S5D). However, the depletion of YTHDF3 significantly inhibited GFP production from circRNA but not from linear mRNA (Supplementary information, Figure S5E and S5F), suggesting that YTHDF3 is essential for circRNA translation driven by m^6^A. Consistently, YTHDF3 can directly interact with eIF4G2 as judged by the reciprocal co-immunoprecipitation assays (Figure 3G), suggesting a possible role of YTHDF3 in recruiting eIF4G2 to the m^6^A containing RNA. Interestingly, compared to canonical translation from linear mRNAs, m^6^A-driven circRNA translation was more sensitive to treatment of hygromycin B (Supplementary information, Figure S6), a well-studied antibiotic that inhibits ribosome translocation during translation elongation^41^, raising the possibility that different modes of translation initiation may affect elongation.

In addition, we examined the possible translation of endogenous circRNA through genomic analyses of the *in vivo* binding sites for various initiation factors (from CLIP-seq data set of ENCODE project, https://www.encodeproject.org), the transcriptome-wide m^6^A profiles^20,21^, and the mapping of translation initiation sites (TIS)^42^. Because CLIP reads across the circular-specific splice junction are too low for a meaningful comparison, we used the total reads that may be contributed by both linear and circRNAs. We computed the frequencies of total mRNAs that are bound by eIF4G2, eIF3A or eIF4G1, and compared them to those of the previously reported circRNAs region^3^. We found that, although circRNAs generally have reduced binding of translation initiation factors compared to mRNAs (Figure 3H, white vs light blue bars), circRNAs containing pre-mapped m^6^A and TIS are about twice as often bound by eIF4G2 and eIF3A compared to mRNAs (Figure 3H, white vs dark blue bars). The observation that eIF4G2 and eIF3A prefer to bind circRNAs with m^6^A and TIS sites is consistent with our findings that these factors promoted circRNA translation (Figure 3B-3F). As a control, we found both types of circRNAs have reduced binding to the initiation factor eIF4G1 that is required for cap-dependent translation, again suggesting that circRNA translation is initiated in a cap-independent fashion. As expected, the binding sites of eIF4G2 often overlap with m^6^A modification near the predicted TIS, as exemplified by the circRNA of E3 ubiquitin-protein ligase ARIH2 (Figure 3I).

### Identification of endogenous circRNAs that contain m^6^A modification

**Figure 4:**
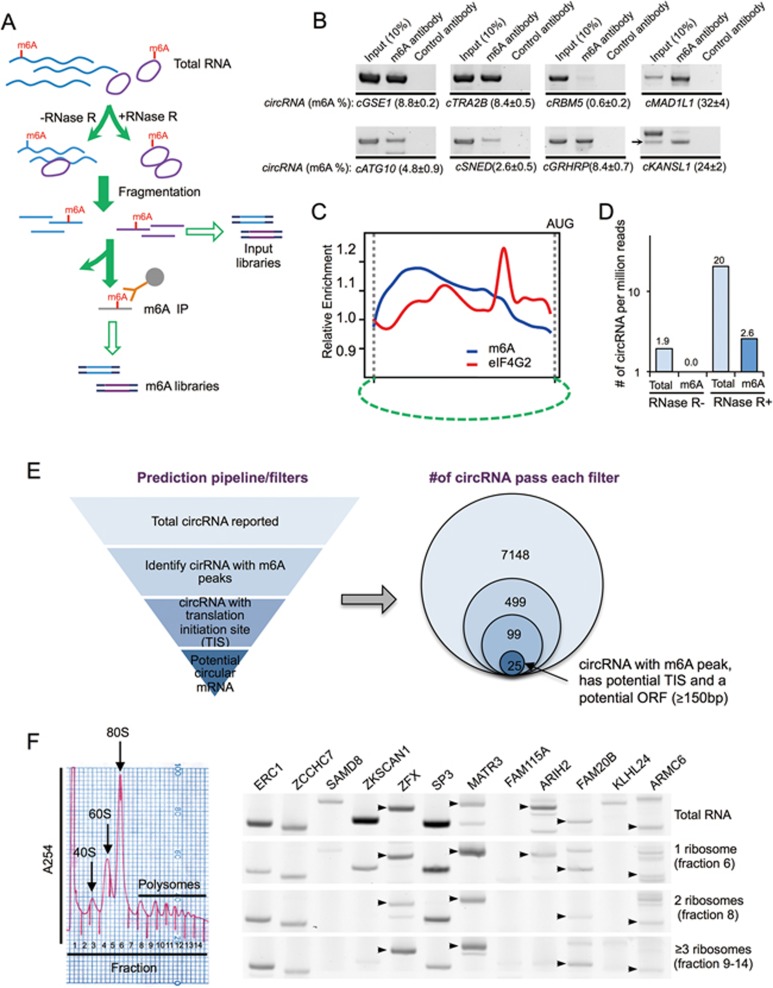
Transcriptome-wide sequencing of m^6^A-modified circRNAs and predictive identification of endogenous circular mRNAs. **(A)** Schematic diagram of circRNA-m^6^A-seq protocol. **(B)** Validation of m^6^A-modified circRNAs in immunoprecipitated samples using m^6^A antibody or control antibody. Arrows indicate predicted circRNA size in the lanes with multiple bands, input (10%) indicates 10% of total input RNAs were used for RT-PCR, % m^6^A indicates percentage of m^6^A modification in target circRNAs (m^6^A antibody/(10× input (10%)). **(C)** Positional distribution of relative density for m^6^A and eIF4G2 binding site in circRNAs as compared to the respective control samples. The putative start codon was used as arbitrary marker to align the plot. When multiple AUG sites are presented in the circRNAs, the AUG that generates the longest ORF is use. **(D)** Number of circRNA reads (i.e., back-splice junction reads) per million of total reads in circRNA-m^6^A-seq samples treated with or without RNase R. **(E)** Schematic diagram of translatable circRNA prediction pipeline. Left, computational filters sequentially applied to identify circRNAs that contain m^6^A site, translation initiation site (TIS) and an ORF with sufficient length. The circRNA, m^6^A and TIS-sequencing data are from published results (see Materials and Methods section). Right, the numbers of circRNAs passing each filters. **(F)** Detection of predicted circRNAs from various host genes in polysome fractions with RT-PCRs. In the lanes with multiple bands, the circRNAs with expected size are indicated with arrows. IP, immunoprecipitation.

To assess the importance of m^6^A-mediated translation of circRNAs in cells, we conducted parallel sequencing to identify the m^6^A-containing endogenous circRNAs (circRNA-m^6^A-seq) using m^6^A immunoprecipitation of the RNA samples treated with exoribonuclease RNase R (Figure 4A). We mapped the RNA reads from both input and m^6^A-IP samples, and defined circRNAs according to the reads that span a back-splice junction. We identified 85 circRNAs (supported by 2 450 back-splicing junction reads) with m^6^A as judged by m^6^A-IP (Supplementary information, Table S2). We further tested eight circRNAs by RT-PCR with primers across back-spliced junction, and confirmed that all circRNAs tested are enriched by m^6^A-antibody precipitation compared to treatment with control antibody (Figure 4B). By comparing to the circRNA levels in the input sample, we found that circRNA-m^6^A-seq is very sensitive and can detect circRNAs with low m^6^A modification rate (0.6% m^6^A modification rate in cRBM5, Figure 4B). In addition, compared to the read density obtained using control antibodies, both m^6^A sites and eIF4G2-binding sites were relatively enriched in circRNAs that contain a putative start codon AUG (Figure 4C) (see Materials and Methods section). Intriguingly, the m^6^A displayed a broad peak located upstream of eIF4G2-binding sites, supporting a potential functional cooperation between these two elements in driving circRNA translation.

On the basis of the number of circRNA reads recovered from m^6^A IP vs the total input circRNA reads sequenced, we estimate that ∼13% of total circRNAs had the m^6^A modification (Figure 4D, 2.6/20=13%). This is probably a conservative estimate due to our stringent experimental design and data analysis, in which only a fraction of m^6^A-containing RNAs were precipitated and only the fragments containing m^6^A sites adjacent to the back-splice junction were recovered as positive circRNA reads (Figure 4A). Nevertheless, these data suggest that circRNAs are extensively modified by m^6^A.

### circRNAs with coding potential are common in human transcriptome

On the basis of the above observations, we developed a computational pipeline to predict endogenous translatable circRNAs using a series of filters (Figure 4E). Starting from a trustable set of 7 771 circRNAs previously discovered via sequencing of RNAs resistant to RNase R from Hs68 cells^3^, we first identified 623 circRNAs containing m^6^A peaks (as judged by m^6^A-seq, from HEK293 cells)^20,21^, and further reduced these candidates to 124 circRNAs using the pre-mapped TIS (from HEK293 cells) as a filter^42^. Finally we applied an arbitrary filter of ORF length and selected only those 25 circRNAs with a sufficiently long ORF (≥ 150 nt or encoding a protein longer than 50 aa; Figure 4E). Further validation with polysome profiling confirmed that 10 out of 12 tested circRNAs from selected 25 circRNAs were indeed associated with polysomes, with the circRNA from KLHL24 being the only clear negative (Figure 4F). The other circRNA, cFAM115A, was not detected in total RNAs, presumably because it is not expressed in the cell line we tested (Figure 4F). We also used cMART3 and cARL67P1 as examples to examine the circRNA distribution in the entire polysome gradient. We found that cMART3 was present in all fractions including monosome- and polysome-bound fractions (Supplementary information, Figure S7). As a negative control, cARL67P1, a circRNA that did not pass our computational filters, was not associated with polysome (fractions 8-14) (Supplementary information, Figure S7). As we started with a small trusted data set of 7 700 circRNAs from human fibroblasts, this prediction pipeline is expected to have low sensitivity but high specificity. In addition, because of the incomplete coverage of m^6^A-seq and the limited number of pre-mapped TIS, together with the different cell lines used in existing data, we expect that the 25 circRNAs obtained through these stringent filters only represent a very small fraction of all circRNAs with coding potential in cultured cells. Future studies in a more consistent cellular context will likely increase the sensitivity of detection of translatable circRNAs.

**Figure 5:**
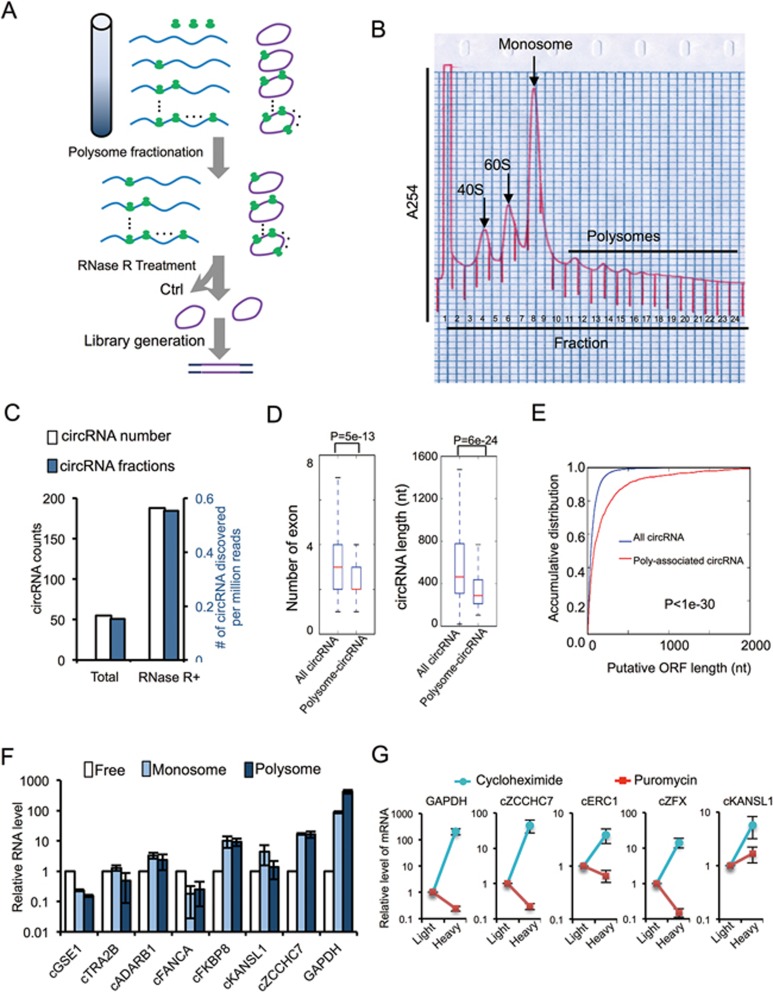
Systematic discovery of circular mRNA in human cells. **(A)** Schematic diagram of polysome-bound circRNA-seq protocol. **(B)** Polysome fractionation of HeLa cell lysate. All Fractions were collected. Fraction 8 was marked as R1, fraction 11 was marked as R2 and fraction 13-20 were combined together and marked as R3. Total RNA from R1, R2 and R3 were isolated separately. **(C)** Numbers and frequencies of circRNA junction reads detected by polysome-bound circRNA seq in samples with or without RNase R treatment. **(D)** Comparison of the number of exons and the length between polysome-associated circRNAs and the total circRNAs. All *P*-values were calculated with KS-test. **(E)** Accumulative distribution of the length for putative ORFs in polysome-associated circRNAs and the total circRNAs. **(F)** Relative ratios of monosome- and polysome-bound RNAs vs unbound (free) RNAs for several circRNAs. The linear mRNA of GAPDH was used as control. **(G)** HeLa cells were treated with 200 μM puromycin or cycloheximide, lysed and separated by sucrose gradient centrifugation. The RNAs from light fractions (< 60S) and heavy fraction (> 2 ribosomes) were purified and used as template for real-time RT-PCR reactions. The relative levels of RNAs associated with the heavy fraction vs the light fraction were plotted for each circRNA or GAPDH mRNA.

The above findings inspired us to experimentally identify the circRNAs undergoing active translation in human transcriptome. We first used sucrose gradient centrifugation to purify polysome-associated RNAs, and subsequently treated the purified samples with RNase R and subjected them to high-throughput sequencing (Figure 5A). The resulting reads were mapped to human genome using CIRCexplorer to identify back-splicing junctions^8^. We identified 250 circRNAs that are associated with polysomes (Figure 5B; Supplementary information, Table S3), with ∼0.6 circRNA per million total reads in RNase R-treated samples (Figure 5C). It is worth noting that this result was obtained again using stringent criteria because only the polysome-associated fragments containing back-splice junctions were considered as circRNA reads (see Materials and Methods section). As a result, only 1 out of 25 translatable circRNA predicted from above pipeline was recovered in the unbiased polysome profiling/circRNA-seq, suggesting that our methods are far from saturation. When comparing with all circRNAs, we found that polysome-associated circRNAs tend to have fewer exons and are generally shorter (Figure 5D). On the other hand, polysome-associated circRNAs have longer putative ORFs as compared to all circRNAs in general (Figure 5E), consistent with their expected coding potential. This result also suggests that a larger fraction of polysome-associated circRNAs accounts for putative ORFs.

We further examined the monosome- and polysome-associated circRNAs using quantitative RT-PCR (Figure 5F), and confirmed that all seven tested circRNAs were indeed associated with ribosomes, with five out of seven having more RNAs associated with ribosomes than with the unbound fraction. Moreover, the distribution of circRNAs showed greater bias towards monosomes than polysomes compared to the highly expressed GAPDH mRNA that was mainly bound to polysomes (Figure 5F), suggesting a possibly slower initiation process. Nevertheless, for some circRNAs, a large fraction was associated with ribosomes (e.g., > 20-fold more circRNAs are bound by ribosomes in cFKBP8 and cZCCHC7), suggesting that they are under active translation. In addition, the polysome-associated circRNAs (i.e., heavy fractions) were significantly reduced upon treatment of cells with the puromycin that specifically disrupts active translating ribosomes (Figure 5G, compare the treatment with cycloheximide vs puromycin), suggesting that association of circRNAs with polysomes is likely due to active translation. Taken together, our observations demonstrate that m^6^A-containing circRNAs with coding potential are widespread in human transcriptome.

### Endogenous peptide translated from circular mRNA junction

To directly identify endogenous proteins encoded by circRNAs, we generated a customized database containing peptides encoded by RNA sequences spanning back-splice junctions of all known circRNAs^43^, and combined this peptide database with all human proteins from UniProt to search tandem mass spectrometry (MS/MS) data from total lysates of 293 cells. Our search was performed against a database that includes reversed entries, which minimizes the false discovery rate from the random noise of MS/MS data.

**Figure 6:**
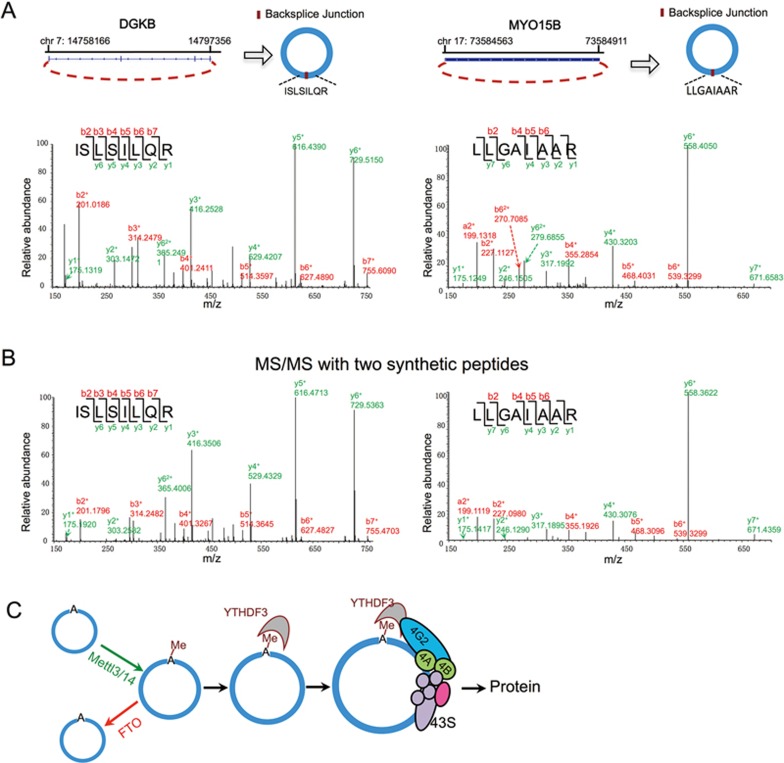
Identification of circRNA junction-coded peptides. **(A)** The collision-induced dissociation (CID) MS/MS spectrum of the [M+2H]^3+^ ion at *m/z* 465.29 of the human cDGKB peptide ISLSILQR and of human cMYO15B peptide LLGAIAAR ([M+2H]^2+^ ion at *m/z* 392.75) shown as an example. Annotated b- and y-ions are listed above and below the peptide sequence marked in red and green color, respectively. **(B)** The CID spectrum of MS/MS for corresponding synthetic peptides match to human cDGKB peptide ISLSILQR and cMYO15B peptide LLGAIAAR were shown as confirmation for the product of circRNA translation. Annotated b- and y-ions are listed above and below the peptide sequence marked in red and green color, respectively. **(C)** A schematic diagram of circRNA translation driven by m^6^A.

We identified 33 peptides (19 unique peptide in total, some being identified multiple times in replicated samples) encoded by the back-splice junctions of circRNAs that do not match any known proteins from UniProt (Supplementary information, Table S4, sheet 1). The collision-induced dissociation MS/MS spectrum from two representative examples was shown (Figure 6A). To further validate candidate circRNA-encoded peptides, we chemically synthesized these two circRNA-encoded peptides and used them to re-run the MS/MS analysis. The collision-induced dissociation MS/MS spectrum from both synthesized peptides closely matched those of the original peptide identified from cell lysate (comparing Figure 6B with 6A), suggesting that the peptides identified by proteomic analyses are likely produced from circRNA translation. These peptides merely represent a small fraction of the circRNA-encoded proteome, because only the peptide sequence spanning the back-splice junction can be unambiguously identified as circRNA-encoded products. However, we did not find any functional enrichment of the host genes of these circRNAs despite circRNA translation being elevated by cellular stress, probably because of the limited coverage of this method.

## Discussion

In this report, we serendipitously found that a variety of sequences can function as IRESs to drive circRNA translation, and also observed that m^6^A is responsible for the promiscuous circRNA translation (Figure 1). We further demonstrated that circRNAs contain extensive m^6^A modifications, which are sufficient to drive protein translation in a cap-independent fashion involving the m^6^A reader YTHDF3 and the translation initiation factors eIF4G2 and eIF3A (Figure 6C). Consistently, many circRNAs were found to be associated with polysomes, suggesting that a sizable fraction of endogenous circRNAs (but not all circRNAs) is indeed translated. Searches for polysome-associated circRNAs were previously attempted with conventional approaches, but no significant hits were identified^3,14,15^. Our approach has increased sensitivity by starting with a large amount of raw material, using RNase R treatment to enrich circRNAs, and sequencing more than 700 million reads. Although we only identified a small number of circRNA-encoded peptides due to the stringent filters used in MS/MS analyses (unique mapped peptides across back-splicing site), m^6^A modifications are very common in circRNAs as judged by circRNA-m^6^A-seq, suggesting that translatable circRNAs may be common in human transcriptome. These results challenge the stereotypic view of circRNAs as non-coding RNAs, and open new paradigms for potential function of circRNAs.

This finding leads to many intriguing questions. For example, what are the possible functions of circRNA-encoded proteins? We found that circRNA translation is increased under heat shock condition, raising the possibility that circRNA-encoded proteins may play roles in stress response. It has been proposed that cap-independent translation through IRESs is increased in cancers to promote translation of genes that play important roles in stress responses, development, apoptosis and cell cycle regulation^44^. Because circRNA can only be translated through cap-independent translation, we speculate that the translation from circRNA may be more prevalent in cancer cells, a question to be addressed in future. In addition, many circRNAs code for N-terminal protein fragments, potentially generating protein isoforms that have overlap sequences with conventionally tranlated protein. As a result, it is possible that the circRNA-coded isoforms can interfere with the function of the respective canonical protein. Interestingly, we also found that several short sequences without consensus m^6^A motifs (RRACH) can also drive translation in our circRNA reporter (data not shown), implying either that circRNA translation could also be driven through some m^6^A-independent mechanisms, or alternatively the methylation of adenosine may occur in non-canonical sites.

Our findings have some fundamental implications: While most circRNAs may be classified as non-coding RNAs, some of them likely function as mRNAs because they contain m^6^A and TIS, and are associated with polysomes, blurring the definition of coding and non-coding RNAs. Previously some non-coding RNAs are reported to have coding potential from 5′ ORFs^45,46^, however the extensive cap-independent translation driven by m^6^A *in vivo* suggests an even more pervasive translation of non-coding RNAs from internal ORFs. Therefore, the translational landscape of a cell may be much more complicated than currently appreciated, i.e., the alternative ORFs commonly found in mRNAs may indeed be translated by internal m^6^A sites. It was generally known that many mass spectrum peaks cannot be reliably assigned to known proteins in proteomic studies and this was mainly attributed to existence of unknown modifications. However, alternative reading frames of mRNA may contribute to some of the unassigned peaks. Just as one gene can produce multiple mRNA isoforms through alternative splicing, we speculate that extensive cap-independent translation may enable one mRNA to be translated into multiple proteins.

## Materials and Methods

### Plasmid construction, cell culture and transfection

The circRNA reporters containing split GFP^11^ were inserted with different human endogenous IRES, control sequences and putative m^6^A motifs using *EcoR*I and *EcoR*V cloning sites in the reporter (see Supplementary information, Table S1 for inserted sequences). The expression vector for FTO was constructed by cloning HA-tagged FTO cDNA into pcDNA5/FRT/TO using *Nhe*I and *Kpn*I sites. The pcDNA3-Flag-METTL3 and pcDNA3-Flag-METTL14 plasmids were obtained from Addgene, and the pcDNA3-Flag-eIF4G2 expression vector is the generous gift from Dr Nahum Sonenberg.

293 and HeLa cells were cultured with DMEM medium containing 10% of FBS. To transiently express circRNA reporter, 293 cells were plated into 24-well plates 1 day before transfection. Of note, 1 μg of the plasmids was transfected using lipofectamine 2000 according to the manufacturer's manual. Transfected cells were collected 48 h after transfection for further RNA and protein analysis. For co-transfection, the circRNA reporter was transfected with protein overexpression plasmids in ratio 1:3.

### Semi-quantitative RT-PCR and real-time PCR

Total RNAs were isolated using TRIZOL reagent and treated with DNase I (37 °C, 1 h, followed by heat inactivation). For semi-quantitative PCR, 2 μg total RNA was reverse-transcribed with SuperScript III (Invitrogen), and one-tenth of the RT product was used for PCR (22 cycles, supplemented with trace amount of Cy5-dCTP). The products were separated on 10% PAGE gels, scanned with a Typhoon 9400 scanner, and quantified with ImageQuant 5.2 or stained by SYBR Green I (Thermo Scientific). The real-time PCR was performed using the Maxima SYBR Green qPCR Master Mix (Thermo Scientific) and a 7500 real-time PCR system (Life Technologies) according to the manufacturer's instructions.

### Western blot

Cells were lysed in buffer containing 50 mM HEPES, 150 mM NaCl, 1 mM EDTA, 1% (w/v) CHAPS and Sigma protease inhibitor cocktail, and the total cell lysates were resolved with SDS-PAGE gels. The following antibodies were used: GFP antibody (632381) from Clontech; GAPDH antibody (sc-32233) from Santa Cruz; Flag antibody (F1804) from Sigma; HA antibody (SC-805) from Santa Cruz. HRP-linked secondary antibodies were used and blots visualized with the ECL kit (Bio-Rad).

### Gene knockdown with lentiviral shRNA

shRNA plasmids were purchased from the TRC library through GE Dharmacon. shRNA plasmids were transfected into 293 cells with psPAX2 and pMD2.G in ratio 4:3:1. Virus was collected at 48 h after transfection. 293 cells were infected by the lentivirus for 48 h followed by 2 μg/ml puromycin selection.

### m^6^A immunoprecipitation and quantification

Total RNAs were isolated from cells and treated with DNase I (37 °C, 1 h, followed by heat inactivation). 20 μg total RNA was incubated with 2 μg anti-m^6^A antibody (Synaptic Systems 202003) or GAPDH antibody in 200 μl IP buffer (10 mM Tris-HCl, 150 mM NaCl, 0.1% (vol/vol) Igepal CA-630, 2 mM ribonucleoside vanadyl complexes (Sigma-Aldrich) and 0.5 U/μl RNasin (Promega)) for 2 h at 4 °C. During the incubation, the protein A/G PLUS-agarose beads (Santa Cruz) were blocked by IP buffer supplemented with BSA (0.5 mg/ml) for 2 h at 4 °C, washed three times in 500 μl IP buffer, and then mixed with the total RNAs/anti-m^6^A antibodies in IP buffer (2 h at 4 °C). After the incubation, beads were washed three times with 500 μl IP buffer, and bound RNAs isolated with TRIZOL reagents. Recovered RNA was then analyzed by real-time RT-PCR.

### Polysome fractionation and sequencing

HeLa cells were pre-treated with 200 μM cycloheximide for 5 min at 37 °C and washed with ice-cold PBS containing 200 μM cycloheximide. Cells were then lysed with polysome lysis buffer (400 mM KOAc (pH 7.5), 25 mM K-HEPES, 15 mM Mg(OAc)_2_, 1 mM DTT, 200 μM cycloheximide, 1% NP-40, 0.5% deoxycholate, 1 mM PMSF and 50 U/ml RNasin) for 10 min on ice. Cell debris was removed by centrifugation at 14 000 rpm for 10 min at 4 °C, and the supernatant was loaded onto 10-ml continuous 15-50% sucrose gradients containing 400 mM KOAc (pH 7.5), 25 mM K-HEPES, 15 mM Mg(OAc)_2_, 200 μM cycloheximide and 50 U/ml RNasin. The samples were centrifuged at 4 °C for 3 h at 100 000× *g* in an SW41 rotor (Beckman), and the fractions were collected using a Brandel Fractionation System and an Isco UA-6 ultraviolet detector used to produce polysome profiles for gradients. Total RNA was extracted from each fraction by TRIZOL.

Ribosomal RNA was depleted from these fractionated RNAs by RiboMinus Human/Mouse Transcriptome Isolation Kit. Half of the recovered RNA was treated with RNase R at 37 °C for 1 h followed by ethanol precipitation. The purified RNA was used for library preparation with KAPA-stranded RNA-seq kit.

### Analysis of m^6^A motifs in circRNAs

The circRNA data set was derived from a previous study^3^ and introns removed based on annotation from circBase (http://www.circbase.org/). For comparison, we also analyzed total mRNAs, which were separated into coding sequences, exons, introns, transcription start sites, transcription termination sites, start codons and stop codons based on Refseq gene annotation. We determined the frequency of m^6^A motif (HRRACH, H=A/C/T, R=A/G) based on counts of these motifs normalized by the length of certain region. As control, we calculated the average frequency of 1 000 random 6-mers in the circRNA data set.

### Percent of binding site of eIF4G2, eIF3A and eIF4G1

We defined circRNAs including m^6^A peaks^20^ and ribosome binding sites^47^ as potential coding circRNAs. Using CLIP-seq data sets of eIF4G2 and eIF4G1 from the ENCODE project (https://www.encodeproject.org), and eIF3A from a published data set^32^, we computed the percent of these factors' binding sites contained in mRNAs, circRNAs and potential coding circRNAs.

### Analysis of the density of m^6^A-seq peaks and CLIP-seq data, and circRNA

The pre-mapped m^6^A-seq reads were downloaded from previous datasets^20,21^, and the reads number calculated using sliding 20 nt windows along the full-length circRNA. The CLIP-seq data were downloaded from ENCODE. We calculated the mean coverage of a specific region for each window of the immunoprecipitated samples and controls. To calculate the enrichment of signals in each window, the coverage of the IP samples (m^6^A or eIF4G2) was normalized by the mean coverage of entire gene, then divided by normalized coverage of corresponding window in control samples.

### Polysome- and m^6^A-associated circRNA detection

We detected circRNA using CIRCexplorer pipeline. First reads were aligned to GRCh37 human genome with Tophat, and then unmapped reads were realigned with Tophat-Fusion. Finally, back-spliced junction reads were annotated with Refseq gene annotation.

### Mass spectrometry detection of circRNA-coded proteins

Proteins were precipitated with trichloroacetic acid. The protein pellet was dried either by air or by using a Speedvac for 1-2 min. The pellet was subsequently dissolved in 8 M urea, 100 mM Tris-HCl, pH 8.5. TCEP (final concentration is 5 mM) (Thermo Scientific) and iodoacetamide (final concentration is 10 mM) (Sigma) for reduction and alkylation were added to the solution and incubated at room temperature for 20 and 15 min, respectively. The protein mixture was diluted four times and digested with Trypsin at 1:50 (w/w) (Promega, http://www.promega.com/).

For multidimensional protein identification technology (MudPIT), total peptide mixtures were pressure-loaded onto a biphasic-fused silica capillary column. The entire column setting (biphasic column-union-analytical column) was placed in line with an Agilent 1200 quaternary HPLC pump (Palo Alto, CA, USA) for MS analysis. The digested proteins were analyzed using an eight-step MudPIT separation method as described previously^48^.

A back-splice junction database was constructed based on circBase^43^, from which the circRNA sequences were extracted using BEDTools^49^ using hg19 annotation of human genome. The peptides spanning the back-splice junctions were translated in all reading frames from 5′ to 3′. We combined all human protein sequences from UniProt and back-splice junction databases as a customized database to search the spectra. Peptides obtained from MS/MS across back-splice site were also used to search non-redundant human protein database with BALSTP to ensure that these peptides are not from any known human protein. The acquired MS/MS data were analyzed against the customized protein database using Protein Discoverer 2.0 (Thermo Scientific). Mass tolerances for precursor ions were set at 20 ppm and for MS/MS were set at 0.8 Da. Trypsin was defined as cleavage enzyme with three most miss cleavage, the mass of the amino acid cysteine was statically modified by + 57.02146 Da, the FDR was set at 0.01 for protein identification by searching against a database that includes reversed entries.

See Supplementary information, Data S1 for detailed methods.

## Author Contributions

YY and ZW designed the study and prepared the manuscript. YY, MM and XS conducted experiments, and XF conducted the computational analyses. PW and CW conducted the mass spectrometry analyses. YZ and LC helped in detecting circRNA with northern blot. YY, JY, YW and XX helped to analyze the data and provided comments in paper revision.

## Competing Financial Interests

The authors declare no competing financial interests.

## Supplementary Material

Supplementary information, Table S4

Supplementary information, Figure S1 circRNA is translated by m^6^A motif

Supplementary information, Figure S2 circRNA validation by northern blot and RNase R treatment.

Supplementary information, Figure S3 Translation of circRNA in different cell types.

Supplementary information, Figure S4 eIF4G2 and eIF3A knockdown affects global protein synthesis rate

Supplementary information, Figure S5 m^6^A reader protein affect translation of circRNAs

Supplementary information, Figure S6 m^6^A driven circRNA translation is more sensitive to treatment of hygromycin B.

Supplementary information, Figure S7 Entire expression profile across the polysome gradient.

Supplementary information, Data S1 Supplementary experimental procedures
