# Supplementary material for: Extensive translation of circular RNAs driven by N6-methyladenosine
Source: Cell Res. 2017 Mar 10;27(5):626–41. doi: 10.1038/cr.2017.31 (PMC5520850; doi:10.1038/cr.2017.31)
Supplement: Supplementary information, Figure S1 — circRNA is translated by m6A motif [file cr201731x5.pdf]

Supplemental Figures

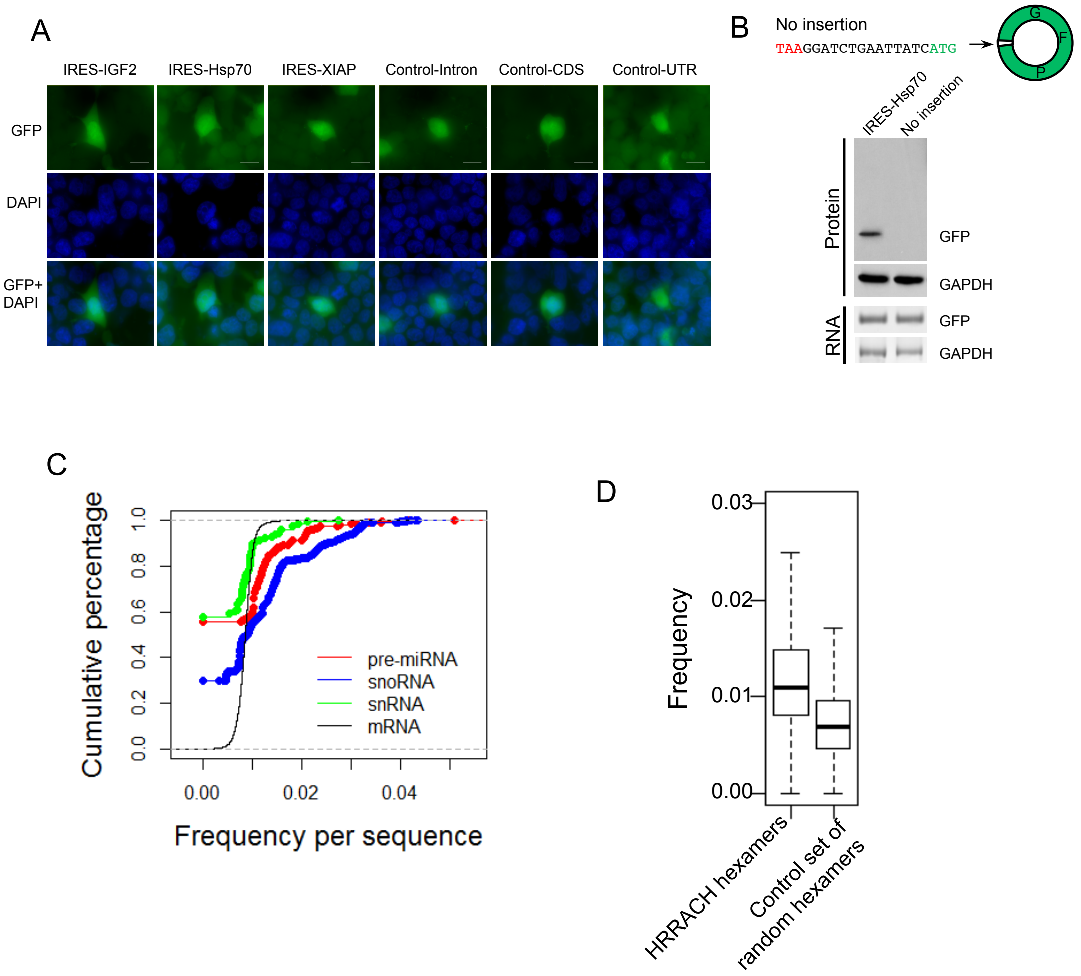

Figure S1. circRNA is translated by m⁶A motif

(A) 293 cells were transiently transfected with circRNA translation reporters containing different endogenous human IRESes (from IGF2, Hsp70 and XIAP) or three control sequences (short fragments of intron, coding region and 5'-UTR from beta-Actin gene, see table S1). The cells were assayed by fluorescence microscopy 48h after induction. Scale bar = 30 µm.

(B) The true negative control for circRNA translation. The non-insertion reporter is generated by EcoRI/EcoRV digestion, followed by DNA polymerase I-Klenow fragment treatment to fill the overhang site and then self-ligation. The translation from resulting reporter was undetectable.

(C) Accumulative distribution of m^6A motif in different types noncoding RNAs and mRNAs.

(D) Frequency of m^6A motif (HRRACH) and random generated 1000 sets of control hexamers (36 hexamers in each set) in circRNA. The frequency is defined as occurrence number of hexamers normalized by sequence length. P-value < 2.2e-16 (KS-test).
