# Supplementary material for: Extensive translation of circular RNAs driven by N6-methyladenosine
Source: Cell Res. 2017 Mar 10;27(5):626–41. doi: 10.1038/cr.2017.31 (PMC5520850; doi:10.1038/cr.2017.31)
Supplement: Supplementary information, Figure S2 — circRNA validation by northern blot and RNase R treatment. [file cr201731x6.pdf]

A

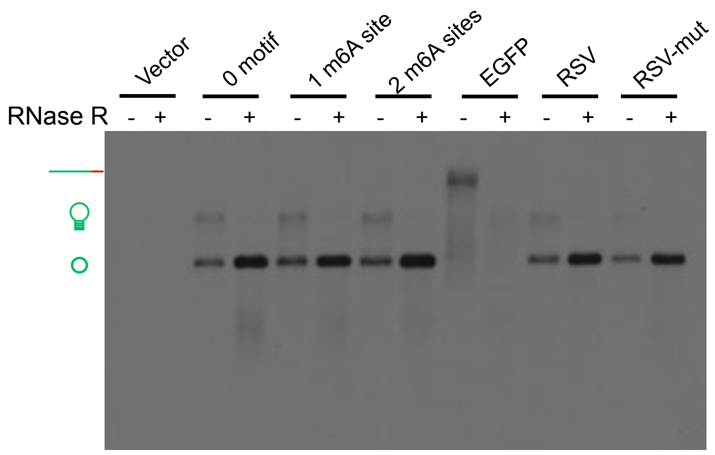

B

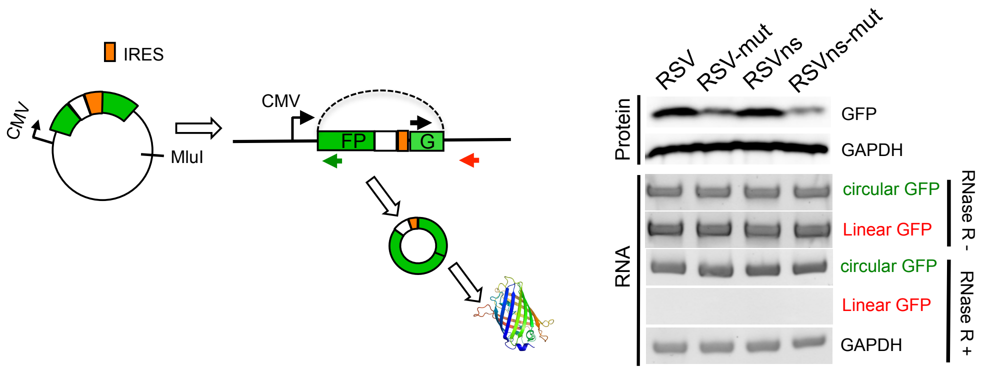

Figure S2. circRNA validation by northern blot and RNase R treatment.

(A) circRNA validation by northern blot. circRNA reporters were transfected into 293 cells. Total RNA was isolated by Trizol, and treated with or without RNase R 48 hours after transfection. DIG labeled full length GFP RNA probe was used to detect circular and linear GFP RNA. Vector indicated empty vector without GFP expression, EGFP indicated EGFP-C1 vector which can express a linear GFP, 0 motif, 1 motif and 2 motif, RSV and RSV-mut indicated circRNA reporter containing different m^6A motif (sequence information see Fig. 1). Green and red linear indicated linear EGFP-C1 RNA, green circle indicated circular GFP RNA, green hair pin indicated folded pre-circular GFP RNA without back-splicing.

(B) Linearized circRNA reporters were tested for the activity of driving translation. circRNA reporters containing RSV, RSVns and their mutations were linearized by MluI. Then linear plasmid was transfected into 293 cells to detect protein production with western blot 48 hours after transfection. Total RNA was treated with or without RNase R to detect circular GFP and linear GFP by RT-PCR.
