# Supplementary material for: Extensive translation of circular RNAs driven by N6-methyladenosine
Source: Cell Res. 2017 Mar 10;27(5):626–41. doi: 10.1038/cr.2017.31 (PMC5520850; doi:10.1038/cr.2017.31)
Supplement: Supplementary information, Figure S3 — Translation of circRNA in different cell types. [file cr201731x7.pdf]

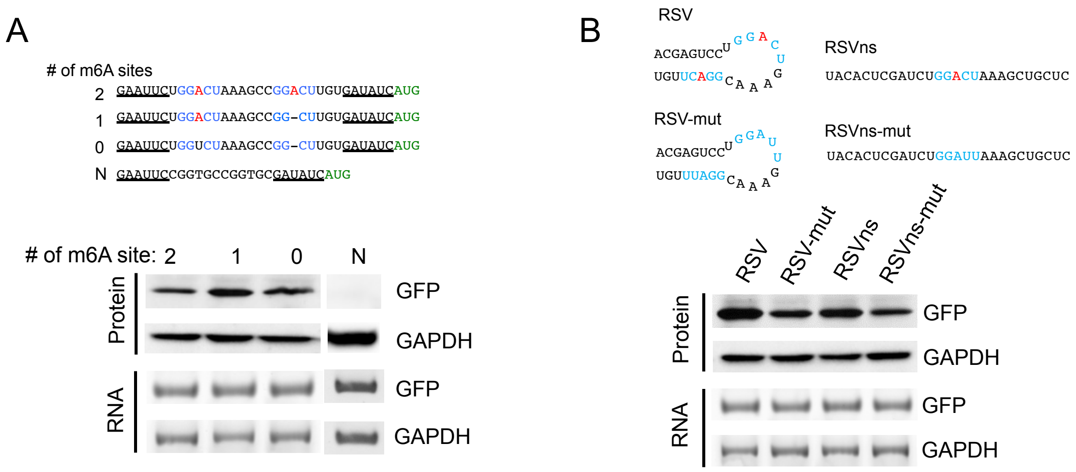

Figure S3. Translation of circRNA in different cell types.

(A) Translation of circRNA can be driven by m^{6}A motifs in HeLa cells. 0-2 copies of m^{6}A motifs (GGACU) were inserted at upstream of AUG in circRNA reporters, and each reporter was transfected into HeLa cells to detect protein production with western blot 48 hours after transfection.

(B) Known m^{6}A sites (RSV and RSVns) and their mutations were tested for the activity of driving translation. Experimental procedure is same as panel a.
