# Supplementary material for: Extensive translation of circular RNAs driven by N6-methyladenosine
Source: Cell Res. 2017 Mar 10;27(5):626–41. doi: 10.1038/cr.2017.31 (PMC5520850; doi:10.1038/cr.2017.31)
Supplement: Supplementary information, Figure S4 — eIF4G2 and eIF3A knockdown affects global protein synthesis rate [file cr201731x8.pdf]

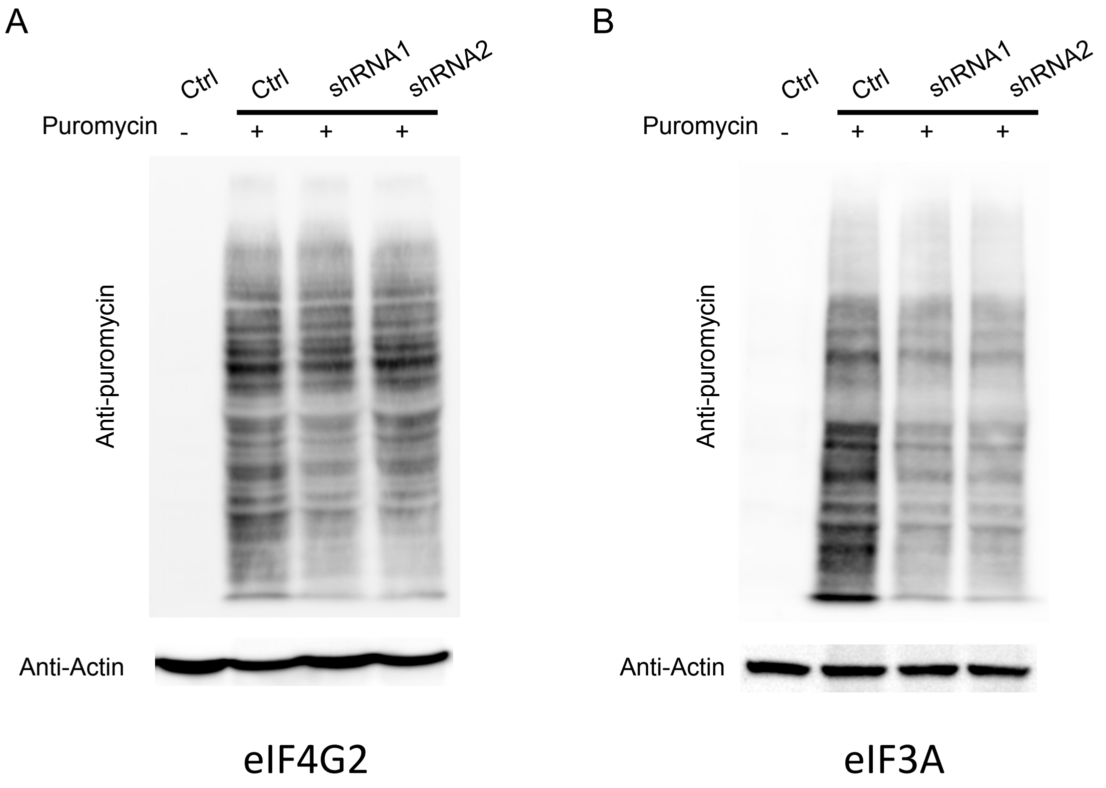

Figure S4. eIF4G2 and eIF3A knockdown affects global protein synthesis rate

(A) eIF4G2 knockdown slightly decreased global protein synthesis rate. Cells were treated with 1μg/ml puromycin for 30 min. Total protein were resolved with SDS-PAGE gels and transferred from the gel to PVDF membrane. Puromycin labeled nascent proteins were probed by anti-puromycin antibody.

(B) eIF3A knockdown significantly decreased global protein synthesis rate. Experimental procedure is same as panel a.
