# Supplementary material for: Extensive translation of circular RNAs driven by N6-methyladenosine
Source: Cell Res. 2017 Mar 10;27(5):626–41. doi: 10.1038/cr.2017.31 (PMC5520850; doi:10.1038/cr.2017.31)
Supplement: Supplementary information, Figure S5 — m6A reader protein affect translation of circRNAs [file cr201731x9.pdf]

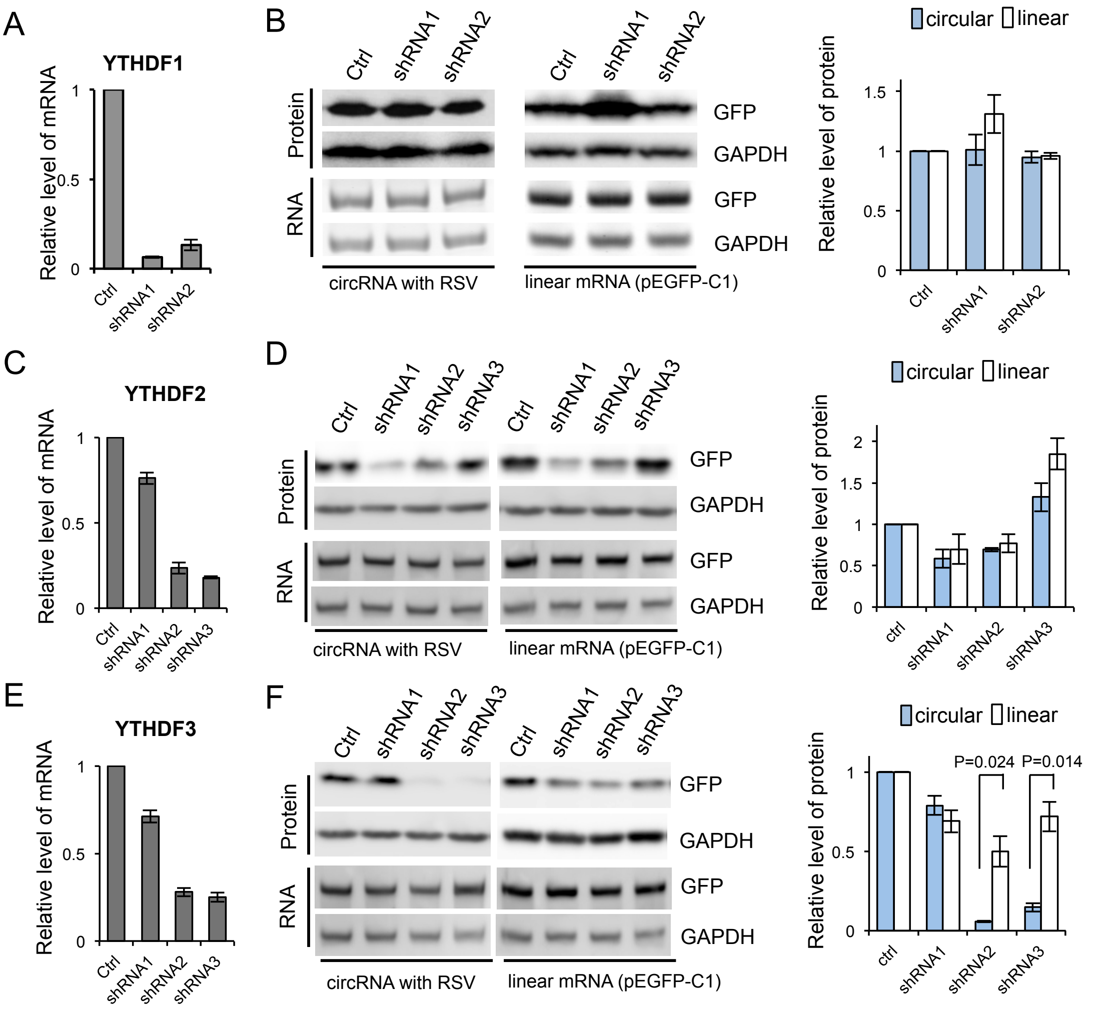

Figure S5. m^{6}A reader protein affect translation of circRNAs

(A) YTHDF1 knockdown by two different shRNAs stably expressed in 293 cells, mRNA levels were measured by realtime RT-PCR.

(B) RNAi of YTHDF1 does not affect circRNA translation. HEK293 cells stably expressing shRNAs of YTHDF1 were transfected with circRNA reporters containing RSV sequence or linear GFP reporters (pEGFP-C1). RNA and protein expression were analyzed by semi-quantitative RT-PCR and western blots (left). Quantification of GFP protein levels normalized to GAPDH (right), and the relative value to mock transfection control was plotted (n=3, Mean±SD).

(C) YTHDF2 knockdown by three different shRNAs stably expressed in 293 cells. Only two of the three shRNAs have significantly reduced mRNA level.

(D) HEK293 cells stably expressing shRNAs of YTHDF2 were transfected with GFP expression vector of circular or linear RNA reporters. The experimental condition and quantification is same as panel b.

(E) YTHDF3 knockdown by three different shRNAs stably expressed in 293 cells.

(F) HEK293 cells stably expressing shRNAs of YTHDF3 were transfected with GFP expression vector of circular or linear RNA reporters. The experimental condition and quantification is same as panel b, p values were calculated with paired t test.
