# Supplementary material for: Extensive translation of circular RNAs driven by N6-methyladenosine
Source: Cell Res. 2017 Mar 10;27(5):626–41. doi: 10.1038/cr.2017.31 (PMC5520850; doi:10.1038/cr.2017.31)
Supplement: Supplementary information, Figure S6 — m6A driven circRNA translation is more sensitive to treatment of hygromycin B. [file cr201731x10.pdf]

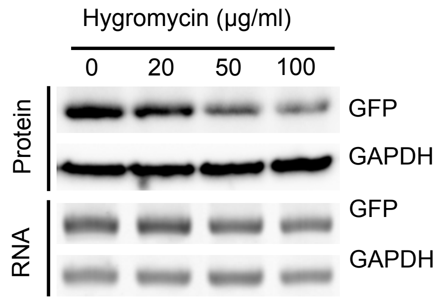


**Figure S6. m$^6$A driven circRNA translation is more sensitive to treatment of hygromycin B.** 293 cells were transfected with circRNA reporters containing RSV sequence. The media was changed to fresh media with 0, 20, 50, 100μg/ml hygromycin after 6 hours. After 48hrs, the cells were collected, and RNA and protein expression were analyzed by RT-PCR and western blots.
