# Supplementary material for: Extensive translation of circular RNAs driven by N6-methyladenosine
Source: Cell Res. 2017 Mar 10;27(5):626–41. doi: 10.1038/cr.2017.31 (PMC5520850; doi:10.1038/cr.2017.31)
Supplement: Supplementary information, Figure S7 — Entire expression profile across the polysome gradient. [file cr201731x11.pdf]

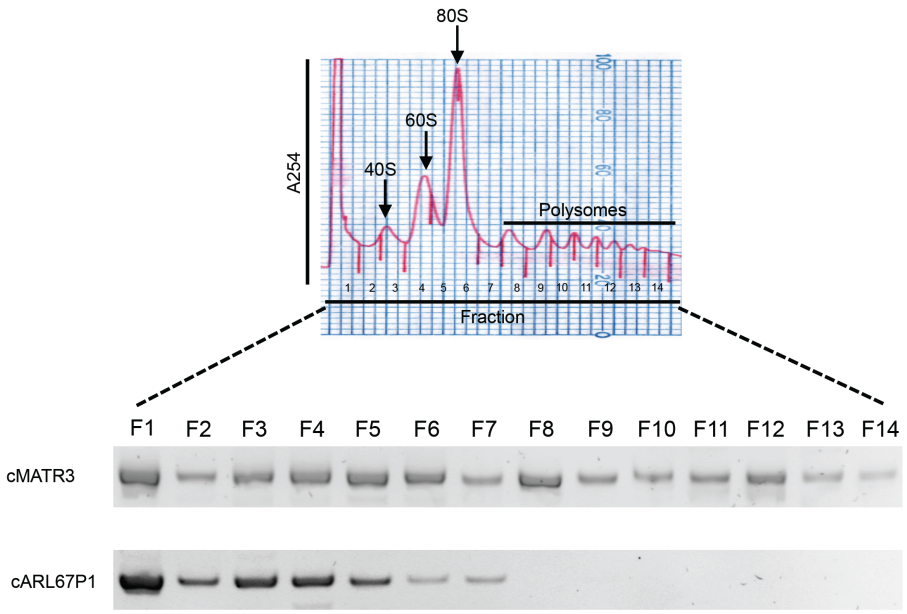

Figure S7. Entire expression profile across the polysome gradient. HeLa cells were treated with 200 µM cycloheximide, lysed and separated by sucrose gradient centrifugation. The RNAs from different fractions (F1-F14) were purified and used as template for RT-PCR reactions. PCR product was separated by 6% PAGE gel and stained by SYBR Green I.
