# Supplementary material for: Extensive translation of circular RNAs driven by N6-methyladenosine
Source: Cell Res. 2017 Mar 10;27(5):626–41. doi: 10.1038/cr.2017.31 (PMC5520850; doi:10.1038/cr.2017.31)
Supplement: Supplementary information, Data S1 — Supplementary experimental procedures [file cr201731x12.pdf]

Supplementary information, Data S1: supplementary experimental procedures

Plasmid construction, cell culture and transfection

The circRNA reporters containing split GFP was constructed previously [1]. The EMCV IRES was replaced by different human endogens IRES, control sequences and putative m^{6}A motifs using EcoRI and EcoRV cloning sites in the reporter (see table S1 for inserted sequences). We also generated a non-insertion reporter by EcoRI/EcoRV digestion, followed by DNA polymerase I-Klenow fragment treatment and then self-ligation of blunt ends. The expression vector for FTO was constructed by cloning HA-tagged FTO cDNA into pcDNA5/FRT/TO using NheI and KpnI sites. The pcDNA3-Flag-METTL3 and pcDNA3-Flag-METTL14 plasmids were obtained from Addgene, and the pcDNA3-Flag-eIF4G2 expression vector is the generous gift from Dr. Nahum Sonenberg.

293 and HeLa cells were cultured with DMEM medium containing 10% of FBS as described previously [1]. To transiently express circular RNA reporter, 293 cells were plated into 24-well plates 1 d before transfection. Of note, 1 μg of the plasmids were transfected using lipofectamine 2000 according to the manual. Transfected cells were collected 48hrs after transfection for further RNA and protein analysis. For co-transfection, circRNA reporter was transfected with protein overexpression plasmids in ratio 1:3.

Semi-quantitative RT-PCR and real-time PCR

Total RNAs were isolated using TRIZOL reagent and treated with DNase I (37 ℃, 1 h, followed by heat inactivation). For semi-quantitative PCR, 2μg total RNA was reverse-transcribed with SuperScript III (Invitrogen), and one-tenth of the RT product was used for PCR (22 cycles, supplemented with trace amount of Cy5-dCTP). The products were separated on 10% PAGE gels, scanned with a Typhoon 9400 scanner, and quantified with ImageQuant 5.2 or stained by SYBR Green I (Thermo Scientific). The real-time PCR was performed using the Maxima SYBR Green qPCR Master Mix (Thermo Scientific) and a 7500 real-time PCR system (Life Technologies) according

to manufacturer′s instructions.

Western blot

Cells were lysed in buffer containing 50 mM HEPES, 150 mN NaCl, 1 mM EDTA, 1% (w/v) CHAPS and Sigma protease inhibitor cocktail, and the total cell lysates were resolved with SDS-PAGE gels. The following antibodies were used: GFP antibody (632381) from Clontech; GAPDH antibody (sc-32233) from Santa Cruz; Flag antibody (F1804) from sigma; HA antibody (SC-805) from Santa Cruz. The HRP-linked secondary antibodies were used and the blots were visualized with the ECL kit (Bio-Rad).

Northern blot

Total RNAs were isolated using TRIZOL reagent and treated with DNase I (37 ℃, 1 h, followed by heat inactivation). Half of DNase I treated RNA was treated with RNase R at 37 ℃ for 1hr following ethanol precipitation. Same amounts of RNAs with or without RNase R treatment were separated by agarose gel, and were transferred from gel to nylon membrane. Then RNAs were immobilized with UV cross-linking an Ultraviolet Crosslinker (UVP, CL-1000, 254-nm wavelength). Dig (Digoxigenin)-labeled RNA probes were prepared by RiboMAX™ Large Scale RNA Production Systems (Promega) according to the manufacturer’s instructions. The membrane was pre-hybridized by DIG Easy Hyb (3 ml/100 cm^2) for 30 mins at 68 ℃, and hybridized by denatured DIG-labeled RNA probe (100 ng/ml) overnight. Then the membrane was washed twice with 2X SSC, 0.1% SDS for 5 mins and twice with 0.2X SSC, 0.1% SDS for 30 mins, and blocked by 1X blocking buffer. Finally the DIG-labeled RNA probe was detected by anti-Digoxigenin-AP, Fab fragments (Roche, 11093274910).

Gene knockdown with lentiviral shRNA

shRNA plasmids were purchased from the TRC library through GE Dharmacon. shRNA plasmids were transfected into 293 cells with psPAX2 and pMD2.G in ratio

4:3:1. Virus was collected at 48hrs after transfection. 293 cells were infected by the lentivirus for 48hr followed by 2μg/ml puromycin selection.

Global protein synthesis assay using puromycin labeling

Knockdown and control Cells were cultured with DMEM medium containing 10% of FBS without puromycin two days before assay. Cells (at 40-50% confluence) were treated by 1μg/ml puromycin for 30 mins. Then cells were harvested and lysed by RIPA buffer, and the total cell lysates were resolved with SDS-PAGE gels. The total proteins were transferred from the gel to PVDF membrane. Puromycin labeled nascent proteins were probed by anti-puromycin antibody (from kerafast, EQ0001). The HRP-linked secondary antibodies were used and the blots were visualized with the ECL kit (Bio-Rad).

m^6A immunoprecipitation and quantification

Total RNAs were isolated from cells and treated with DNase I (37 ℃, 1 h, followed by heat inactivation). 20μg total RNA was incubated with 2μg anti- m^6A antibody (Synaptic Systems 202003) or GAPDH antibody in 200 µl IP buffer (10 mM Tris-HCl, 150 mM NaCl, 0.1% (vol/vol) Igepal CA-630, 2mM Ribonucleoside vanadyl complexes (Sigma-Aldrich) and 0.5U/µl RNasin (Promega)) for 2 hrs at 4°C. During the incubation, the protein A/G PLUS-agarose beads (Santa Cruz) were blocked by IP buffer supplemented with BSA (0.5 mg/ml) for 2 hrs at 4°C, washed 3 times in 500µl IP buffer, and then mixed with the total RNAs/anti- m^6A antibodies in IP buffer (2 hrs at 4 ℃). After the incubation, beads were washed 3 times by 500µl IP buffer, and the bound RNAs were isolated with TRIZOL reagents. Recovered RNA was then analyzed by real time RT-PCR.

Co-immunoprecipitation

HEK293 cells were cultured in 10 cm dishes one day before transfection, and different combinations of Flag-eIF4G2/HA-YTHDF3, Flag-eIF4G2/empty vector, or empty vector/HA-YTHDF3 were co-transfected into HEK293 cells by lipofectamine

2000. Two day after transfection, cells were washed by cold PBS twice, and then lysed in 1000 µl buffer containing 20 mM Tris HCl (PH=8.0), 137 mM NaCl, 10% glycerol, 1% Nonidet P-40, 2mM EDTA and Sigma protease inhibitor cocktail. Cells lysate were incubated on ice 30mins, and then centrifuge for 10 mins at 12,000 rpm, 4 ℃. Supernatant was transferred to new tube. 1µg anti-Flag or anti-HA antibody was added into 250 µl cell lysate, and then incubate sample overnight at 4°C, under agitation. Then 50 µl washed protein A/G PLUS-agarose beads (Santa Cruz) were added into samples, and incubated 2 hours at 4 ℃, under agitation. After the incubation, beads were washed 3 times by 1000µl IP buffer, and resuspended with 1X SDS loading buffer, incubated at 95 ℃ for 5 min before SDS-PAGE separation and western blotting.

circRNA m^{6}A**-seq**

Ribosomal RNA was depleted from total RNA by RiboMinus™ Human/Mouse Transcriptome Isolation Kit (Thermo Fisher) followed by ethanol precipitation. Half of the recovered RNA was treated with RNase R (epicentre) at 37 ℃ 1 hr followed by ethanol precipitation. The RNA was then chemically fragmented into 100bp by 8 min incubation at 94 ℃ in fragmentation buffer (40mM Tris-OAc, 100mM KOAc, 30 mM $Mg(OAc)_2$, pH 8.3). The fragmented RNA was used for m^{6}A immunoprecipitation as described above. After washing, bound RNA was eluted from beads with 0.5 mg/ml N^6-methyladenosine (Sigma-Aldrich) in IP buffer followed by ethanol precipitation. The purified RNA was used for library preparation with KAPA stranded RNA-seq kit.

Polysome fractionation and sequencing

HeLa cells were pre-treated with 200 µM cycloheximide for 5 min at 37 ℃ and washed by ice-cold PBS containing 200 µM cycloheximide. Cells were then lysed with polysome lysis buffer (400 mM KOAc (pH 7.5), 25 mM K-HEPES, 15 mM $Mg(OAc)_2$, 1mM DTT, 200 µM cycloheximide, 1% NP-40, 0.5% deoxycholate, 1mM PMSF and 50 units/mL RNasin) for 10 min on ice. Cell debris was removed by centrifugation at 14,000 rpm for 10 min at 4 ℃, and the supernatant was loaded onto

10-mL continuous 15%–50% sucrose gradients containing 400 mM KOAc (pH 7.5), 25 mM K-HEPES, 15 mM $Mg(OAc)_2$, 200 μM cycloheximide and 50 units/mL RNasin. The samples were centrifuged at 4 ℃ for 3 hrs at 100,000*g* in an SW41 rotor (Beckman), and the fractions were collected using a Brandel Fractionation System and an Isco UA-6 ultraviolet detector used to produce polysome profiles for gradients. Total RNA was extracted from each fraction by TRIZOL.

Ribosomal RNA was depleted from these fractionated RNAs by RiboMinus™ Human/Mouse Transcriptome Isolation Kit. Half of the recovered RNA was treated with RNase R at 37 ℃ for 1hr followed by ethanol precipitation. The purified RNA was used for library preparation with KAPA stranded RNA-seq kit.

Analysis of m^6A **motifs in circRNAs**

circRNA dataset was derived from previous study [2] and intron was removed based on annotation from circBase (http://www.circbase.org/). For comparison, we also analyzed total mRNAs, which were separated into coding sequences (CDS), exon, intron, transcription start site, transcription termination, start codon, and stop codon based on Refseq gene annotation. We determined the frequency of m^6A motif (HRRACH, H=A/C/T, R=A/G) based on counts of these motifs normalized by the length of certain region. As control, we calculated the average frequency of 1000 random 6-mers in circRNA dataset.

Percent of binding site of eIF4G2, eIF3A, and eIF4G1

We defined circRNAs include m^6A peak [3] and ribosome binding site [4] as potential coding circRNAs. Using CLIP-seq datasets of eIF4G2 and eIF4G1 from ENCODE project (https://www.encodeproject.org), and eIF3A from published dataset [5], we computed the containing percent of these factors' binding sites in mRNA, circRNA, and potential coding circRNA.

Analysis of the density of m^6A **-seq peaks and CLIP-seq data and circRNA**

The pre-mapped m^6A -seq reads were downloaded from previous dataset [3,6], and we

calculated the reads number using sliding windows of 20nt windows along the full length circRNA. The CLIP-seq data was downloaded from ENCODE. We calculated the mean coverage of specific region for each window of the immunoprecipitated samples and controls. To calculate the enrichment of signals in each window, the coverage of the IP samples (m^6A or eIF4G2) was normalized by the mean coverage of entire gene, then divided by normalized coverage of corresponding window in control samples.

Polysome and m^6A **associated circRNA detection**

We detected circRNA using CIRCexplorer pipeline. First reads were aligned to GRCh37 human genome with Tophat, and then unmapped reads were realigned with Tophat-Fusion. Finally, back spliced junction reads were annotated with Refseq gene annotation.

Mass Spectrometry Detection of circ-RNA coded Proteins

Proteins were precipitated with trichloroacetic acid (TCA) using the following protocol: to 1 volume of sample solution (cold), add 1/3 volume of 100% (w/v) TCA (6.1N, Sigma), mix well to give a final TCA concentration of 25%, leave on ice for 3 hrs. Spin for 30 min at 4 ℃, aspirate the supernatant, leave 5−10 µL in the tube so as not to disturb the pellet. Wash twice with ice-cold acetone (500 µL each). After each wash, spin for 10 min. The protein pellet was dried either by air or by using a Speedvac for 1−2 min. The pellet was subsequently dissolved in 8 M urea, 100 mM Tris-HCl, pH 8.5. TCEP (final concentration is 5 mM) (Thermo Scientific) and Iodoacetamide (final concentration is 10 mM) (Sigma) for reduction and alkylation were added to the solution and incubated at room temperature for 20 and 15 minutes, respectively. The protein mixture was diluted four times and digested with Trypsin at 1:50(w/w) (Promega, http://www.promega.com/).

MudPIT LC-MS/MS Analysis

For multidimensional protein identification technology (MudPIT), total peptide

mixtures were pressure-loaded onto a biphasic fused-silica capillary column (250 μm i.d.) consisting of 2.5 cm long strong cation exchange (5 μm partisphere, Whatman, Clifton, NJ) and 2.5 cm long reversed phase (Aqua C18, Phenomenex, Torrance, CA), which was prepared by slurry packing using an in-house high pressure vessel. The column was washed with buffer A (see below) for more than 10 column volumes (75 μm i.d.) with a through-hole union (Upchurch Scientific, Oak Harbor, WA). The analytical columns were pulled beforehand by a laser puller (Sutter Instrument Co., Novato, CA) with a 5 μm opening. It was packed with 3 μm reversed phase (Aqua C18, Phenomenex, Torrance, CA) to 15 cm long. The entire column setting (biphasic column-union-analytical column) was placed in line with an Agilent 1200 quaternary HPLC pump (Palo Alto, CA) for mass spectrometry analysis. The digested proteins were analyzed using an 8-step MudPIT separation method as described previously [7].

The buffer solutions used were as follows: water/acetonitrile/formic acid (95:5:0.1, v/v/v) as buffer A, water/acetonitrile/formic acid (20:80:0.1, v/v/v) as buffer B, 500 mM ammonium acetate as buffer C. The elution gradient of step 1 was as follows: 5 min of 100% buffer A, a 5 min gradient from 0 to 10% buffer B, an 80 min gradient from 10 to 45% buffer B, a 10 min of 100% buffer B, a 1min gradient from 100% to 0% buffer B, a 9min gradient of 0% buffer B. Steps 2−7 had the following profile: 1 min of 100% buffer A, 4 min of X% buffer C, followed by the same gradient as step 1. The 4 min buffer C percentages (X) were 10, 20, 40, 60, 80, and 100% respectively. The salt pulse for the final step (step 8) was 90% buffer C plus 10% buffer B (450 mM ammonium acetate in 12.5% acetonitrile).

A back-splice junction database was constructed based on circBase [8], from which the circRNA sequences were extracted using BEDTools [9] using hg19 annotation of human genome. The peptides spanning the backsplice junctions were translated in all reading frames from 5' to 3' direction. We combined all human protein sequence from UniProt and back-splice junction database as a customized database to search the spectra. Peptides obtained from MS/MS across back-splice site were also used to search non-redundant human protein database with BALSTP to ensure that these peptides are not from any known human protein. The acquired MS/MS data were

analyzed against the customized protein database using Protein Discoverer 2.0 (Thermo Scientific). Mass tolerances for precursor ions were set at 20 ppm and for MS/MS were set at 0.8 Da. Trypsin was defined as cleavage enzyme with 3 most miss cleavage, the mass of the amino acid cysteine was statically modified by +57.02146 Dalton, the FDR was set at 0.01 for protein identification by searching against a database that includes reversed entries.

Supplemental References

1 Wang, Y. & Wang, Z. Efficient backsplicing produces translatable circular mRNAs. *Rna* **21**, 172-179, doi:10.1261/rna.048272.114
2 Jeck, W. R. *et al.* Circular RNAs are abundant, conserved, and associated with ALU repeats. *Rna* **19**, 141-157, doi:10.1261/rna.035667.112
3 Meyer, K. D. *et al.* Comprehensive analysis of mRNA methylation reveals enrichment in 3' UTRs and near stop codons. *Cell* **149**, 1635-1646, doi:10.1016/j.cell.2012.05.003
4 Wan, J. & Qian, S. B. TISdb: a database for alternative translation initiation in mammalian cells. *Nucleic acids research* **42**, D845-850, doi:10.1093/nar/gkt1085 (2014).
5 Meyer, K. D. *et al.* 5' UTR m(6)A Promotes Cap-Independent Translation. *Cell* **163**, 999-1010, doi:10.1016/j.cell.2015.10.012
6 Dominissini, D. *et al.* Topology of the human and mouse m6A RNA methylomes revealed by m6A-seq. *Nature* **485**, 201-206, doi:10.1038/nature11112
7 Washburn, M. P., Wolters, D. & Yates, J. R., 3rd. Large-scale analysis of the yeast proteome by multidimensional protein identification technology. *Nature biotechnology* **19**, 242-247, doi:10.1038/85686 (2001).
8 Glazar, P., Papavasileiou, P. & Rajewsky, N. circBase: a database for circular RNAs. *Rna* **20**, 1666-1670, doi:10.1261/rna.043687.113 (2014).
9 Quinlan, A. R. & Hall, I. M. BEDTools: a flexible suite of utilities for comparing genomic features. *Bioinformatics* **26**, 841-842, doi:10.1093/bioinformatics/btq033 (2010).
